# Supplementary material for: High prevalence of falsely declaring nicotine abstinence in lung transplant candidates
Source: PLoS One. 2020 Jun 18;15(6):e0234808. doi: 10.1371/journal.pone.0234808 (PMC7302701; doi:10.1371/journal.pone.0234808)
Supplement: S1 Table — (DOCX) [file pone.0234808.s002.docx]

**Table S1**. **Comorbidities of the study cohort.**

|  | **All (n=620)** |
| --- | --- |
| Arterial hypertension, n (%) | 222 (35.8) |
| Diabetes mellitus, n (%) | 108 (17.4) |
| Hyperlipoproteinemia, n (%) | 66 (10.6) |
| Coronary artery disease, n (%) | 66 (10.6) |
| Osteoporosis, n (%) | 91 (14.7) |
| Gastroesophageal reflux disease, n (%) | 111 (17.9) |
| Atrial fibrillation, n (%) | 33 (5.3) |
| Psychiatric disorder*, n (%) | 80 (12.9) |
| Depression, n (%) | 48 (7.7) |
| Drug or alcohol abuse in the past, n (%) | 23 (3.7) |
| Anxiety/panic disorder, n (%) | 12 (1.9) |
| Pulmonary hypertension, n (%) | 167 (26.9) |
| Mean PAP ± SD (mmHg)^a^ | 26.5 ± 12.4 |

Data are presented as number and percentage, respectively. PAP; pulmonary arterial pressure (mean ± standard deviation). ^a^ Available in 383 patients. *3 patients had concomitant psychiatric disorders.
