# Supplementary material for: High prevalence of falsely declaring nicotine abstinence in lung transplant candidates
Source: PLoS One. 2020 Jun 18;15(6):e0234808. doi: 10.1371/journal.pone.0234808 (PMC7302701; doi:10.1371/journal.pone.0234808)
Supplement: Attachment — Submitted filename: Response to reviewers.docx [file pone.0234808.s003.docx]

Dear Editors, dear Reviewers,

we thank both reviewers and the editor for reviewing our manuscript so carefully and raising important issues that helped us to improve the manuscript. To us, by presenting data in a different format and by clarifying misleading information, the manuscript is now more consistent and gives the reader a more thorough picture of our patients.

Sincerely,

Nikolaus Kneidinger and co-authors

**REVIEWER COMMENTS:**

**The full comments of the reviewers were the following:**

**Reviewer:** #1

**Comments to the Author**

**GENERAL**

The authors used urinary cotinine levels to confirm tobacco abstinence among subjects being evaluated, and/or listed for lung transplantation. They report that active smoking (as evidenced by cotinine levels) is prevalent (~15%) in their lung transplant candidates, and that reliance upon self-reported abstinence is inaccurate for detecting tobacco use.

The study is well done, though I do not believe the study design allows them to faithfully backup their conclusions. Most importantly, though patients are restricted from using nicotine replacement therapy, it is possible (perhaps very likely) that many of the positive urinary screens could be related to individuals self-medicating with over-the-counter nicotine replacement therapy. What the finding of this paper illustrates to me is how profoundly addicting nicotine is, as evidenced by patient factors associated with positive nicotine test (those with psychiatric comorbidities, patients with heavier-pack-year exposure, and more recent quit dates). These factors are known to be associated with both higher prevalence of tobacco use, and greater risk of relapse. It is precisely these individuals, who are more susceptible to nicotine withdrawal symptoms, that might self-medicating with over-the-counter nicotine replacement products in order to avoid relapse to combustible tobacco.

MAJOR Comment:

These individuals should be supported in their attempts at tobacco cessation by any means necessary. Apparently their transplant program prohibits the use of nicotine replacement therapy, which is true of some other programs, but not universally the case for all transplant centers. Apart from the ethical issue of prohibiting use of an effective treatment for an addiction, this prohibition of nicotine replacement therapy probably limits the validity of their conclusions, in that the study as described could not reliably distinguish between surreptitious use of combustible tobacco versus pharmacologic use of NRT. There are alternative biomarkers which, though more expensive, are also more specific to combustible tobacco.

Response: We thank the reviewer for these important comments.

Patients are informed that nicotine addiction is a risk factor for tobacco smoking resumption after transplantation and that nicotine replacement and active tobacco use are equally regarded is a contraindication. In the context of pre-transplant psychiatric evaluation patients are evaluated regarding addictive behaviour and in case non-pharmacological and pharmacological interventions are offered by our institution.

We agree that a clear differentiation between nicotine replacement and active tobacco use is not possible in our study. In total, 92 patients were tested positive and thereof 14 patients truly reported nicotine use (new Figure 1). Thereof, 11 patients reported tobacco and only 3 patients reported to use nicotine replacement products. We cannot conclude that the distribution is similar in patients falsely reporting not using nicotine-based products. However, this could indicate that among positive tested patients there is a considerable number of patients actively using tobacco.

However, we completely agree that this is a limitation; therefor through out the manuscript the assumption of “false reporting smoking” was changed to “false reporting nicotine use” to avoid confusion. Furthermore, the limitation is discussed in the revised version of the manuscript.

In this line, as suggested, we agree with the referee that more sophisticated methods to directly detect active tobacco use in contrast to nicotine replacement products would facilitate patient selection and cessation in patients with addictive behavior. In particular the use of carbon monoxide (CO) or carboxyhemoglobin (COHb) is widely available and could be used to distinguish between active tobacco and nicotine replacement therapy. This is acknowledged in the revised version of the manuscript.

Minor Comment:

the statement in the 2nd to final paragraph of the Results section indicates that the "...number of patients falsely declaring nicotine abstinence increased over the transplant stage" but the data shown in this regard in table 4 do not appear to have appropriate statistical confirmation. While the numbers do increase, the confidence intervals about these averages are likely too large to support a 'trend'.

Response: We thank both reviewers for this important comment. As suggested by referee #2 we performed 2x2 tables with calculations for sensitivity, specificity, negative predictive value (NPV), and positive predictive value (PPV) over different stages prior to lung transplantation (new Figure 3).

Specificity (true negative reports) was high and remained stable over the stages. Sensitivity (true positive reports) was low, and decreased towards transplantation, suggesting that the number of cotinine positive patients falsely declaring nicotine abstinence increased over the stages, towards transplantation. If a patient reports active nicotine use the test is likely positive (PPV) and if a patient declares nicotine abstinence the test is likely negative (NPV).

We agree with the reviewer that the number of patients, in particular of positive tested patients over the stages towards transplantation decrease, therefore, a proper statistical comparison is not possible and interpretation should be performed cautiously. This limitation is acknowledged in the revised version of the manuscript.

**Reviewer:** #2

**Comments to the Author**

Authors present an interesting manuscript on misreporting of smoking status in lung transplant patients. Data have some unique findings and the cohort represents the most recent analysis for this type of cohort. There are several areas where presentation of data in a different format would make information much more useful and clear. Standard calculations for sensitivity, specificity, NPV, and PPV would help as would a flowchart and breakdown of cotinine values. There are suggestions for reanalysis that could make this manuscript much more interesting and valuable, but the value of the data could be very high.

1/. References for urinary and serum cotinine values should be noted.

Response: We thank the reviewer for this request. As suggest, references for urinary and serum cotinine values were added to the revised version of the manuscript.

Jarvis, M.J., et al., Comparison of tests used to distinguish smokers from nonsmokers. *Am J Public Health, 1987*. 77(11): p. 1435-8.

Benowitz, N.L., et al., Optimal serum cotinine levels for distinguishing cigarette smokers and nonsmokers within different racial/ethnic groups in the United States between 1999 and 2004. *Am J Epidemiol*, 2009. 169(2): p. 236-48.

2/. Most of Table 1 is not useful to the manuscript. PFTs could be eliminated and simple description of age, sex, and BMI could be presented as a single line of text. Table 2 is also not particularly helpful and may be more useful as a supplementary table in lieu of suggestions below.

Response: As suggest by the reviewer, the comments are addressed in the revised version of the manuscript. Table 1 was deleted from the manuscript and demographic characteristics are provided in the Results of the manuscript. Table 2 is provided as new Table S1 in the supplement material.

3/. The presentation of results is somewhat confusing. The most interesting findings are in Table 4. It would be much more intuitive if the following were presented perhaps as a flowchart:
Number of patients screened
Number tested
Number with positive cotinine vs. negative cotinine
Number who reported no smoking who tested positive vs. number who reported smoking and tested positive AND number who reported no-smoking who tested negative vs. number who reported smoking who tested negative

Response: We thank the reviewer for this comment. As suggested the results are presented as a flowchart. Since 2016 the German Medical Association request from all German lung transplant centers to provide evidence for nicotine abstinence in lung transplant candidates and suggest to routine use of cotinine measurements. Therefor, from all lung transplant candidates seen in our outpatient clinic from January 2017 until the end of the observation period (June 2019) cotinine tests were available.

New Figure 1

**Figure 1**. Classification according to cotinine results and self-reports. Data are presented as number and percentage, respectively. NR, nicotine replacement.

4/. How many patients reported never smoking according to cotinine results?

Response: In total, 193 patients (31.1%) reported never smoking, defined as a candidate who has never smoked, or who has smoked less than 100 cigarettes in his or her lifetime. All patients were tested negative. Therefore, routine testing in this never smokers seems not to be mandatory. Results are included and discussed in the revised version of the manuscript.

5/. Authors should strongly consider a simple 2x2 table with calculations for sensitivity, specificity, NPV, and PPV according to self-report vs. cotinine positive. Consideration for these according to Stage 1-3 might be interesting as it appears these change closer to waitlisting.

Response: We thank the reviewer for this important comment. We performed 2x2 tables with calculations for sensitivity, specificity, NPV, and PPV over different stages prior to lung transplantation (new Figure 3). Specificity (true negative reports) was high and remained stable over the stages. Sensitivity (true positive reports) was low, however, decreased towards transplantation, suggesting that the number of cotinine positive patients falsely declaring nicotine abstinence increased over the stage, towards transplantation. If a patient reports active nicotine use the test is likely positive (PPV) and if a patient declares nicotine abstinence the test is likely negative (NPV).

The number of patients, in particular of positive tested patients over the stages towards transplantation decrease, therefore, a proper statistical comparison is not possible and interpretation should be performed cautiously.

6/. In the results, the statement “patients with a positive test were more often former smokers (82.6% vs 63.8%, p<0.001)” does not make sense.

Response: We thank the reviewer for this comment and apologize for confusion. We have deleted that part of the statement from the manuscript.

7/. Throughout the manuscript, statements are slightly misleading regarding percentages. For example, in the 6th paragraph of the Results there is a statement “The number of patient falsely declaring nicotine abstinence increased over the transplant stage, with 83%, 86.2% and 90% in stage 1, 2 and 3, respectively.” I believe this should state “The number of cotinine positive patients falsely declaring nicotine abstinence…” These statements need correction throughout including in the abstract.

Response: We thank both reviewers for this important comment and refer to request #5. Changes have been made as suggested throughout the revised manuscript.

8/. In Table 3, annotation “a” is not in the table.

Response: We apologize; the annotation “a” was added in the table (new Table 1).

9/. The binary selection of positive cotinine values provides limited information. A scatterplot of cotinine values for serum and urine over Stage 1-3 would provide significant useful information to see distribution of values change over stage. This would provide an indirect representation of whether cotinine values are generally large, small, or somewhere in between.

Response: Thank you for this comment. As suggest the serum and urine cotinine values of positive tested patients are provided as a scatterplot over the stages in the revised version of the manuscript (Table 2 and new Figure 2).

Whereas medium serum cotinine decreased over the stages, no clear signal was found for urine cotinine. However, the medium cotinine value was lowest in Stage 3 in both, serum and urine. This might indicate that active smoking or nicotine replacement is stopped before follow-up visit in the transplant center in patients active listed for lung transplantation. However, the number of tests, in particular of positive tests is low and decreased over the stages towards transplantation, therefore interpretation should be performed cautiously.

Results and limitations are incorporated and discussed in the revised version of the manuscript.

**Figure 2**. Distribution of cotinine values over stages 1-3. (A) serum levels of cotinine; (B) urinary levels of cotinine. The horizontal lines represent the medium values for each stage.

10/. The last paragraph of the Results is not particularly useful.

Response: Thank you for this comment. The paragraph was deleted from the manuscript.

11/. The final sentence of the abstract is not well supported. From the data, it appears that 85% of patients did not smoke with 81/92/95% of patients not smoking at Stage 1/2/3. Of patients who smoked, the accuracy did not appear to change much during stage. It is possible that patients who were never smokers (not clearly presented here) could be very accurate as would patients who report current smoking. Data suggest that quitting more than 1 year ago resulted in clearly more accuracy than quitting within the past year. Authors need to better consider the data for more representative conclusions.

Response: We agree with the referee that our statement is misleading and not supported by the presented data. As suggested the majority of the patients did report truly not to use nicotine based products and even some patients did report active tobacco use or nicotine replacement products. We agree that there are patients at risk, as assessed by regression analysis (cessation<1 year, high number of pack years, COPD) where regular cotinine tests might be useful. On the other hand, in never-smokers regular cotinine tests seem not to be mandatory. Therefore, our conclusion was revised accordingly and the sentence deleted. To avoid misinterpretation, we clarified that most patients report not to use nicotine based products and were consecutively tested negative. However, even the proportion of patients tested positive (14,8%) was low, the number seems to be high in the context of imminent lung transplantation. Furthermore, of patients tested positive there was a high prevalence of falsely declaring nicotine abstinence (84,8%). Accordingly, title and wording was revised throughout the manuscript.

It is anticipated that the above changes may result in more definitive discussion measures including where measuring cotinine is most useful.
