# Supplementary material for: High prevalence of falsely declaring nicotine abstinence in lung transplant candidates
Source: PLoS One. 2020 Jun 18;15(6):e0234808. doi: 10.1371/journal.pone.0234808 (PMC7302701; doi:10.1371/journal.pone.0234808)
Supplement: Attachment — Submitted filename: Response to reviewers2.docx [file pone.0234808.s004.docx]

Dear Editors, dear Reviewers,

we thank both reviewers and the editor for reviewing our manuscript once more. We have addressed the comments and adopted suggestions accordingly. To us, the manuscript now has improved further and gives the reader a thorough picture of our patients.

Sincerely,

Nikolaus Kneidinger and co-authors

**REVIEWER COMMENTS:**

**The full comments of the reviewers were the following:**

**Reviewer: #1**

Though I am still concerned that the study design does not permit fully addressing the study question, I believe the authors have raised valid points and acknowledged the limitations of their data in support of the conclusions. IN doing so the paper has been strengthened and I support the authors interpretation of their findings.

**Response:** Thank you for your willingness to review and help to improve our manuscript.

**Reviewer #2:**

Results are much clearer and findings are very interesting. From the revised manuscript, the significant finding is that 85% of patients who smoked (or used NRT) did not report this. This is striking and I would offer that the opening to the Discussion could better reflect this finding. This is certainly more interesting than higher rates in COPD or pack years. I would recommend reorganizing the Discussion around this theme and opening with this finding, but otherwise much improved.

**Response:** We thank the referee for his comments to further improve our manuscript. As suggested we reorganized the Discussion accordingly.

In the discussion, paragraph starting with “interestingly, approximately 85% of patients…” this sentence doesn’t make sense. I think the authors are trying to state “Approximately 85% of patients who smoke or use nicotine products do not accurately self-report use, supporting the previous finding that active smoking in underreported by patients with chronic lung disease”

**Response:** Thank you for this comment; we adopted the sentence as suggested.

In the final paragraph of the discussion, the first sentence appears incorrect and could perhaps conclude that while active smoking is present in a minority of patients, there is a high rate of misrepresentation that warrants biochemical confirmation, particularly in patients reporting an ever-smoking history or quitting smoking within the past year.

**Response:** Thank for this suggestion. To avoid misinterpretation, the first sentence of the last paragraph was deleted and replaced by “In conclusion, our data demonstrate that lung transplant candidates who smoke or use nicotine products do not accurately self-report use….”
